# Transcatheter Closure of Patent Ductus Arteriosus in Infants With Weight Under 1,500 Grams

**DOI:** 10.3389/fped.2020.558256

**Published:** 2020-09-22

**Authors:** Alain Fraisse, Carles Bautista-Rodriguez, Margarita Burmester, Mary Lane, Yogen Singh

**Affiliations:** ^1^Pediatric Cardiology Services, Royal Brompton Hospital, London, United Kingdom; ^2^National Heart and Lung Institute, Imperial College London, London, United Kingdom; ^3^Pediatric Cardiac Intensive Care Unit, Royal Brompton Hospital, London, United Kingdom; ^4^Department of Pediatrics—Pediatric Cardiology and Neonatal Medicine, Cambridge University Hospitals NHS Foundation Trust, Cambridge, United Kingdom; ^5^University of Cambridge School of Clinical Medicine, Cambridge, United Kingdom

**Keywords:** patent ductus arteriosus, prematurity, transcatheter closure, patent ductus arteriosus (PDA), extremely preterm infants, percutaneous closure

## Abstract

Persistent patent ductus arteriosus (PDA) is very common in preterm infants, especially in extremely preterm infants. Despite significant advances in management of these vulnerable infants, there has been no consensus on management of PDA—when should we treat, who should we treat, how should we treat and in fact there is no agreement on how we should define a hemodynamically significant PDA. Medical management with non-steroidal anti-inflammatory drugs (NSAIDs) remains the first line of therapy with moderate success rate in closing the PDA. Paracetamol has been reported to be a safe and equally effective medical therapy for closure of PDA. However, additional studies on its long-term safety and efficacy in extremely low birth weight infants are needed before paracetamol can be recommended as standard treatment for a PDA in preterm infants. Surgical ligation of PDA is not without an increased risk of mortality and co-morbidities. Recently, there has been a significant interest in percutaneous transcatheter closure of PDA in preterm infants, including extremely low birth weight infants. Transcatheter PDA closure in preterm ELBW infants is technically feasible with high PDA occlusion success rates and acceptable complication rates as compared to surgical ligation. Many centers have reported promising early- and mid-term follow-up results. However, they need to be further tested in the prospective well-designed studies and randomized controlled trials comparing the results and outcomes of this technique with current treatment strategies including medical treatment before they can be used as the new standard of care for PDA closure in extremely low birth weight infants.

## Indications and Conventional Treatment of a Hemodynamic Significant PDA in Extremely Low Birth Weight Infants

A persistent patent ductus arteriosus (PDA), defined as failure of the ductus to close within 72 h postnatally, is very common in extremely low birth weight infants and seen in around 50% of infants born under 28 weeks of gestation and / or birth weight <1,000 g (1). Preterm infants under 1,500 g are categorized as very low birth weight infants and those under 1,000 g are considered as extremely low birth weight (ELBW) infants (1, 2). The pathophysiology and echocardiographic diagnosis have been covered in the different articles of Frontier's Research topic on “management of PDA in preterm infants” and this article is primarily focused at advances in management *via* transcatheter closure of PDA in preterm infants weighing under 1,500 g. This article is not focused at whether we should treat or not treat PDA but on the feasibility of closing PDA in ELBW infants via transcatheter route when required as an alternative to surgical ligation.

The left to right shunt across a persistent PDA may lead to pulmonary over-circulation and left heart volume-overload (2). Delayed closure of a hemodynamically significant PDA has been reported to be associated with increased risk of bronchopulmonary dysplasia (BPD) / chronic respiratory disease (CLD), prolonged assisted ventilation, necrotizing enterocolitis (NEC), pulmonary hemorrhage, intraventricular hemorrhage (IVH), renal impairment and increased risk of mortality (2–10). This association led to numerous (more than 60) randomized controlled trials (RCTs) trying to prove that the treatment of PDA would be beneficial. However, these trials have failed to demonstrate any improvement in the long term outcomes such as death or BPD / CLD (11). Some trials have reported short term benefits such as early prophylactic use of indomethacin reported reduction of IVH and acute pulmonary hemorrhage and prophylactic surgical ligation reported reduction in NEC (12–14). There are no other proven benefits shown in terms of improvement in long-term outcomes (such as death or BPD) and there are some adverse outcomes [e.g., increased incidence of chronic lung disease was reported in infants treated with ibuprofen (11)].

Despite the reported association between persistent PDA, increased risk of mortality and significant co-morbidities, the treatment strategy remains controversial which could be because of proven benefits from treating the PDA in ELBW infants (11, 15, 16). There is no agreed consensus on management of PDA in preterm infants and there are numerous trials on-going at the moment testing different treatment approaches to placebo or no treatment.

Treatments with non-steroidal anti-inflammatory drugs (NSAIDs) or paracetamol have been used to promote closure when a PDA was found to be hemodynamically significant. NSAIDs, cyclo-oxygenase inhibitors, have moderate success rate in closing the PDA and they may have adverse effects in some infants or even may be contraindicated in presence of certain conditions such as NEC or intestinal perforation and significant renal impairment (17, 18). Medical treatment fails in 20–40% of the cases (19, 20). Following a recent systemic review (2020) by Ohlsson et al., concluded that paracetamol appears to be a promising alternative to indomethacin and ibuprofen for the closure of a PDA with possibly fewer adverse effects. A possible association between prenatal paracetamol and the development of autism or autism spectrum disorder in childhood and language delay in girls has been reported (21). Therefore, additional studies on its long-term safety and efficacy in ELBW infants are needed before paracetamol can be recommended as standard first line treatment for closure of PDA in preterm infants (21).

Surgical ligation of the PDA is often the alternative to failed pharmacological treatment and it is usually performed through left thoracotomy. It is not without the increased risk of mortality and significant morbidities in this vulnerable group of infants. Thirty days mortality rate has been reported around 5–8% (22, 23). Surgical ligation been reported to be associated to post-ligation cardiac syndrome (PLCS), phrenic nerve and vocal cord palsy, pneumothorax, bleeding, and infection (24–26). The post-operative course of preterm infants undergoing surgical ligation of PDA is often complicated by PLCS and despite targeted milrinone prophylaxis, some infants continue to experience hemodynamic instability and postoperative respiratory deterioration (27–30). Studies have also that it may also worsen long-term outcomes, including increasing the risk for bronchopulmonary dysplasia, retinopathy of prematurity and neurosensory impairment, however, controversies remain whether these are related to surgical ligation or prolonged exposure of preterm infants to PDA itself or associated co-morbidities (31–33).

Conservative management approach, consisting of fluid restriction, diuretics and positive end-expiratory pressure, is also being practiced in many neonatal units because of the lack of proven long-term benefits from PDA closure in this population (34–37). Many studies have concluded that conservative management approach is a reasonable option as compared to pharmacologic or surgical treatment (35–41) while some studies have questioned about the safety of this approach because of increased mortality and morbidity (8, 9, 15). Results from RCTs and well-designed prospective studies are awaited to establish risks and benefits of the conservative management approach.

In absence of proven long term benefits from treating PDAs and lack of superiority of one approach to another, currently there is no universal consensus on how best PDA should be managed in preterm infants. However, there is consensus among the neonatal community that well-designed prospective randomized controlled trials of different modalities against placebo are needed, and there are more than 10 large randomized trials of different approaches on-going worldwide (42). This would be interesting to see the results of these trials in the coming years.

Recently there has been an increased interest in transcutaneous closure of PDA, whether this approach can be used as an alternative to surgical ligation or even to medical treatment in the ELBW infants. We describe the background, current evidence and feasibility of this approach in closure of PDA in infants under 1,500 g.

## Transcatheter Closure of PDA in Extremely Preterm Infants: Background

In the most recent years, several cohort studies reported preliminary experience with transcatheter technique, using various devices for PDA closure in premature infants (43–55). Comparison with surgical ligation revealed a positive impact on post procedure pulmonary outcome (49, 50). The procedure has been well-described in preterm infants, including extremely low birth weight (ELBW) infants as small as 640 g (50). Following the results of an ongoing prospective trial using the ADO II AS, this device received FDA and CE approval for transcatheter closure of PDA in premature babies weighting more than 700 g and over 3 days old (51). This has been renamed as Amplatzer Piccolo Occluder® (APO). More recently, a retrospective comparison between surgery and transcatheter closure by Regan et al. reported an increased mechanical ventilation duration during the NICU stay after surgery as compared to infants undergoing transcatheter closure. Additionally, hospital stay could potentially be shorter in cases where transcatheter closure was performed in the 1st month of life (52).

## Patient Selection and Organization of the Procedure

Preterm infants are always referred by neonatologist for percutaneous closure, usually after failure of medical therapy or when medical pharmacotherapy is contraindicated and/or failure to wean from the ventilator. Pre-procedure transthoracic echocardiography confirms the hemodynamic significance (left cavities dilation, functional mitral/aortic regurgitation, and ductal morphology) and transcatheter closure is usually performed in catheterization laboratory. In most centers the procedure is performed in the cardiac catheter lab, although in some centers with portable fluoroscopy equipment the procedure can be performed at the bedside, especially in very unstable patients unsafe to move or when there is no access to cardiac catheterization lab. Transcatheter closure at the bedside under echocardiography guidance-only has also been reported (44). Unfortunately, bedside procedure under echocardiography guidance has to be performed via trans-arterial route (femoral route) without fluoroscopy which carries increased risk of potentially severe life-threatening complications such as limb ischemia and more worryingly it has limited management options for disc embolization if it occurs (56). Hence, despite technical feasibility it is almost never performed on bedside in ELBW infants and smallest reported infant having this procedure successfully done was around 1,400 g. In comparison, when percutaneous PDA closure performed in in the catheter lab it has been performed successfully in infants as small as 700 g (50–52).

## Available Devices

**Figure 1:**
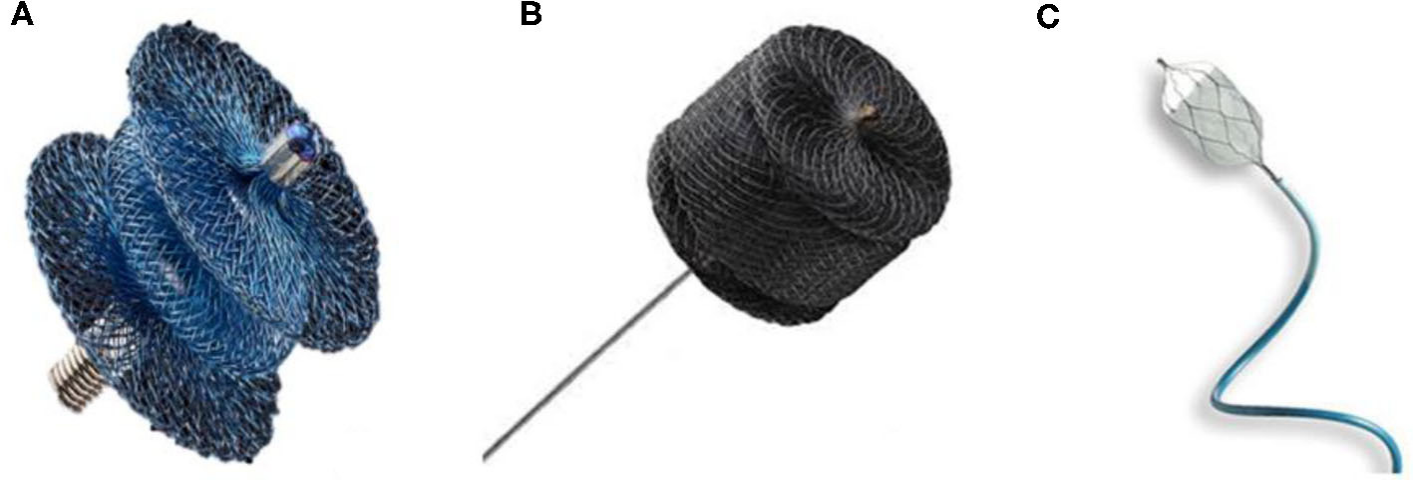
Devices generally used for percutaneous PDA closure in premature babies. **(A)** Amplatzer Ductus Occluder II Additional Sizes / Piccolo Occluder (Abbott). **(B)** Amplatzer vascular plug II (Abbott). **(C)** MVP Microvascular Plug (Medtronic).

Over the last few years, different devices have been used to close PDAs in premature babies (Figure 1). PDA coil-occlusion was initially achieved in selected low-weight infants with symptomatic PDA (44, 51). More recently, the Amplatzer Vascular Plug II (AVP II) was successfully implanted with good results (43, 50, 54). Unfortunately, this device is often not suitable for short ducts in extremely low-weight infants. In such cases the central disk, which is the same diameter than the proximal and distal disk can stretch and make the whole device too long, increasing the risk of LPA and/or aortic stenosis. The Amplatzer Vascular Plug IV has also been used but its length is also a limiting factor in extremely small infants (55). Similarly, the retention disks of the Amplatzer duct occluder II are too large for infants weighing <1,500 g. The Medtronic Micro Vascular Plug® (MVP), initially designed for occlusion of abnormal blood vessels, has shown excellent results for PDA closure in premature babies (47, 50). It is made of a nitinol framework covered partially by a polytetrafluoroethylene (PTFE) membrane at the proximal portion. It is delivered through a microcatheter with two sizes of 5.3 and 6.5 mm. The APO is currently the only dedicated device for this procedure. It is a self-expanding occlusion device with a central waist and retention disc on both ends, delivered through a 4-French delivery catheter. The largest size of the distal disks is 6.5 mm whereas the central waist is available in 3, 4, and 5 mm diameter and the length of the central waist varies between 2, 4, and 6 mm. Catheter and sheath diameters are measured in French (Fr) units and 1Fr = 1/3 mm or 0.013 inch. Wire diameter is measured in inches; 0.014, 0.018, and 0.021 wires are 0.014, 0.018, and 0.021 inch in diameter, respectively (49, 52).

## Description of the Procedure

Ultrasound-guided access of the femoral vein increases the likelihood of successful access and reduces the risk of local complications, especially inadvertent puncture of the femoral artery. Femoral artery access is not recommended and even contraindicated in extremely premature babies due to a high risk for vessel occlusion and potential ischemia of the lower limb (57). Aortic angiogram after device deployment is unnecessary when appropriate echocardiographic guidance can be performed. A 4Fr sheath is usually inserted in the femoral vein.

Heparin administration remains controversial (58). Whereas, some operators give no heparin, others would give 50–100 units/kg of unfractionated heparin bolus once access has been achieved. Prophylactic antibiotics are administered.

Under fluoroscopic guidance, a 3Fr multipurpose BALT catheter (Montmorency, France) is usually advanced over a 0.014 coronary wire through the inferior vena cava toward the right atrium. The coronary wire is advanced *via* right ventricle outflow tract and through the patent ductus arteriosus toward descending aorta. The 3Fcatheter is positioned in the descending aorta. Sathanandam et al. have reported a similar technique using a 4Fr angled glide catheter (Terumo, Japan) and a 0.035 Wholey wire (Medtronic, Minneapolis, MN, USA) to cross the PDA antegradely into the descending aorta (59). Other teams have used 4Fr Swan Ganz catheter instead of catheters described above (43).

**Figure 2:**
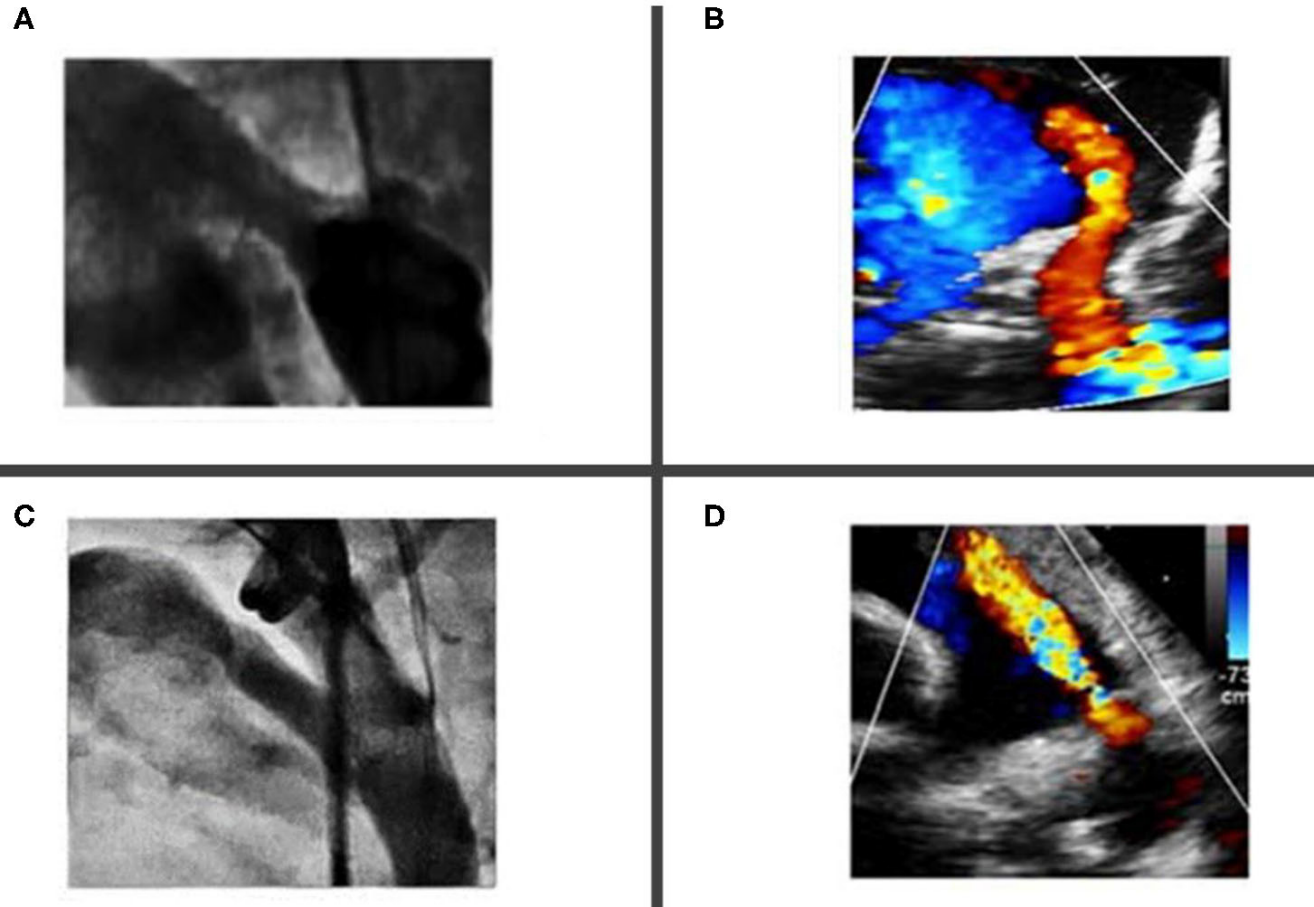
Most common ductal morphology in premature babies undergoing percutaneous closure. **(A,B)** Showing type “C” tubular ductus without any constrictions at the aortic end or the pulmonary artery end. **(C,D)** Showing a “fetal type” ductus, which is typical long, wide and tortuous.

If a 3Fr catheter is used to cross the PDA, then a 0.021 Fixed-Core Guide Wire (Cook Medical®, USA) is advanced in the descending aorta and the 3Fr catheter is directly exchanged with the 4Fr TorqVue® delivery sheath. This latest is often advanced over a microcatheter to minimize mismatch with the 0.021 wire. The tip of the TorqVue® sheath is placed in the descending aorta, slightly lower than the PDA. A hand angiogram of 1.5–2 ml is performed in RAO and lateral projection or only in lateral projection in single plane laboratories. In some centers fluoroscopic guidance only is used (51, 53), although most centers routinely perform this procedure under angiography guidance to minimize the risk of complications. Most of the patients needing PDA closure had type C (tubular) and F (fetal) types of PDA morphology (54) (Figure 2).

**Figure 3:**
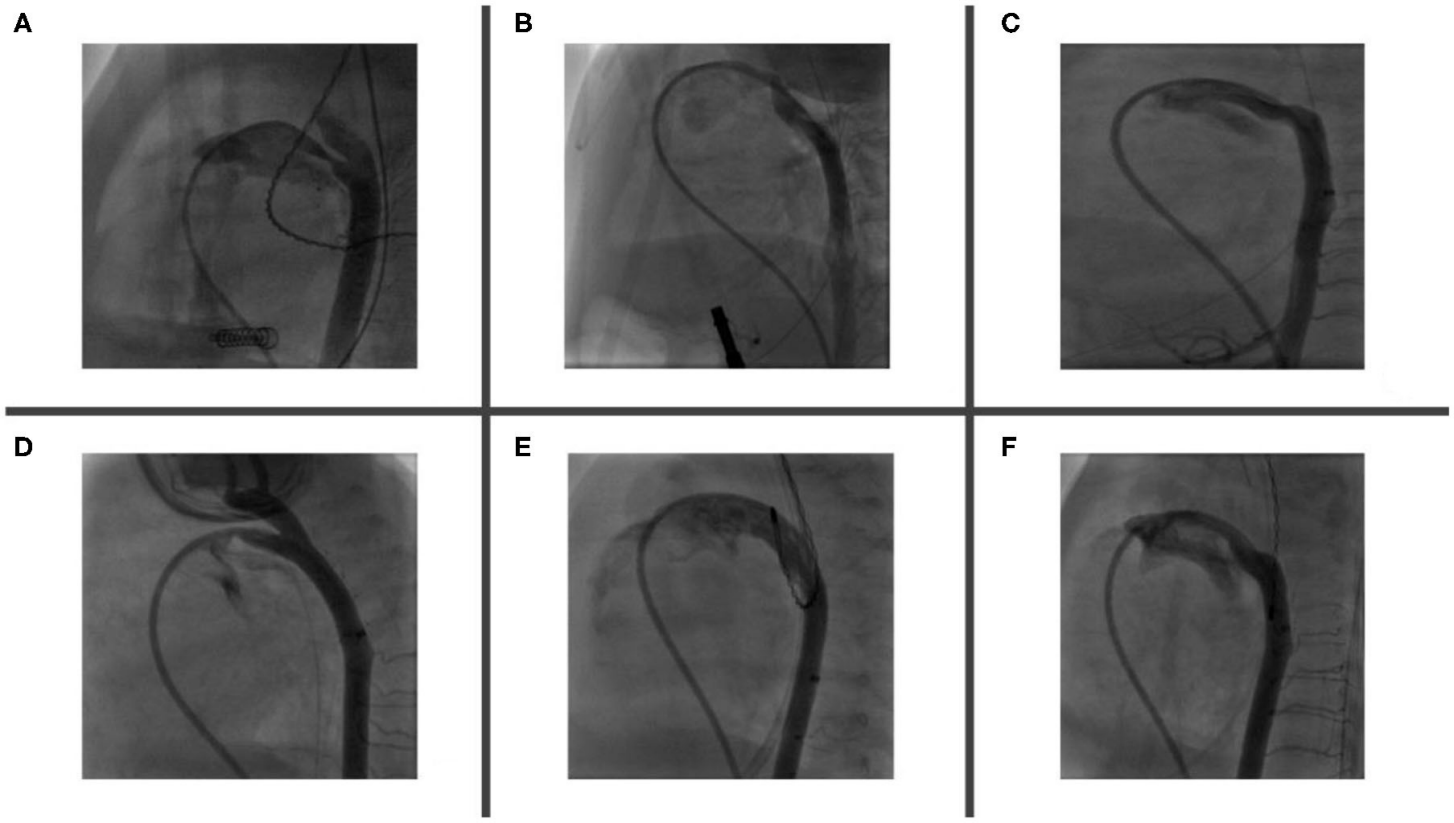
PDA morphology and size are extremely variable in premature babies. Angiogram helps to delineate duct features, accurately measures both PDA's aortic and pulmonary ends and choose the most appropriate device. Angiogram performed through a catheter via RV—MPA—PDA—Descending aorta. Six types of described PDA morphologies have been demonstrated in figures **(A–F)**. **(A)** Relatively long duct with mid-course constriction. **(B)** Long and tortuous duct with pulmonary end constriction. **(C)** Long and tortuous duct with widely open pulmonary and aortic ends. **(D)** Short duct with pulmonary end constriction and LPA origin stenosis. **(E)** Extremely large and dilated duct (note that its diameter is bigger than descending aorta) and **(F)** Long and tortuous duct with variable diameter along its course (RV, right ventricle; MPA, main pulmonary artery; LPA, left pulmonary artery; PDA, patent ductus arteriosus).

The appropriate device is selected based on echocardiographic and angiographic measurements (Figure 3). As a rule, the shortest APO device is used to avoid protrusion of the device in aorta/pulmonary artery branches. In almost all cases only 2 mm or 4 mm-length devices are used.

The device is positioned under fluoroscopy guidance with the goal to implant the entire length of the device into the PDA. Successful positioning is defined by complete occlusion of the duct and the absence of aortic or LPA obstruction by echocardiography.

Following release of the device, echocardiographic evaluation is repeated, paying also particular attention to the tricuspid valve function. Follow-up echocardiography is performed within 24 h after procedure.

## Anesthesia Management

Anesthesia in this high-risk population presents a number of challenges for the anesthesia team. The cardiac catheter laboratory (CCL) can be a hostile environment for these fragile patients. Ambient temperature cannot be easily controlled. The warming blanket should be turned on prior to patient arrival and central patient temperature monitoring is mandatory. Imaging equipment makes patient access during the procedure difficult so patient positioning (arms up) should be optimized prior to allowing equipment in.

Ensure easy access to an intravenous (IV) line, preferably in an upper limb. Two IV lines are essential; one to continue dextrose containing maintenance fluid and the other for fluids and/or drugs required during the procedure. IV access should be secured in the NICU to minimize time in the cardiac catheter laboratory.

Accurate monitoring of end-tidal carbon dioxide (ETCO_2_) can be difficult. It is advisable to have an endotracheal tube (ETT) with minimal leak and minimize circuit dead-space.

There are two periods of potential instability during the procedure however both are generally short-lived and respond quickly to the measures described. Firstly, when the device is positioned in the duct, there is a sudden drop in pulmonary blood flow with a corresponding drop in ETCO_2_ measurements and peripheral saturations. It is advisable to briefly increase the inspired oxygen concentration to maintain acceptable saturation levels until the circulation adjusts. It may also help to temporarily lower the positive end-expiratory pressure to promote forward flow from the right ventricle.

The second is during echocardiography to confirm device position. This can impact significantly on ventilation requiring an increase in ventilatory pressures and the echocardiographer to echo intermittently to ensure adequate ventilation during assessment.

## Reports in the Literature on Venous-Only Access TCPC in Premature Infants

Morville et al. reported successful transcatheter closure of PDA in 32 out of 34 infants under 2,500 g and in their series two patients had major complications: (1) death from hemopericardium and (2) LPA obstruction that required surgical device removal (60). Zahn et al. reported successful PDA closure via transcatheter route in 20 out of 23 premature infants (mean weight 1,250 g; range 755–2,380) and two patients were reported to have complications: (1) LPA stenosis that required stent placement at 3 months of age and (2) transient descending aortic obstruction that was relieved by device manipulation (43, 61). In a small case series 16 patients, Baspinar et al. reported successful closure of PDA in 13 premature infants with a mean weight of 1,000 ± 300 g and three patients were reported to have significant complications including: (1) death from cardiac perforation in one patient and (2) device embolization in two patients, which were retrieved successfully by transcatheter route. These patients needed subsequent surgical closure of the PDA (62). Rodriguez Ogando et al. reported 100% success rate with PDA device closure in 27 preterm infants with a median weight of 1,260 g (range 1,000–1,980) and no major complications were reported (49).

In one of the largest published study so far, Sathanandam et al. reported successful closure of 80 out of 82 preterm infants weighing <2,000 g and three patients were reported to have major complications: (1) death from inferior vena cava laceration, (2) severe LPA stenosis needing manipulation of device, and (3) pericardial effusion needing drainage percutaneously (50).

Recently from one of the largest multi-center study involving 147 infants, 64 undergoing transcutaneous PDA closure or 83 with surgical ligation, Regan et al. reported that transcatheter closure of PDA offers sustained improvement in morbidity as compared to surgical ligation through a reduction in post-procedural mechanical ventilation time that is significant throughout the total NICU stay. They reported successful closure of PDA in 63 out of 64 preterm infants with a mean weight 1,200 g (range 1,025–1,700) and three patients were reported to have complications: (1) severe LPA stenosis needing device retrieval and (2) aortic arch obstruction needing re-intervention (51). Six cases had mild LPA stenoses due to protrusion of the device, with a maximum velocity of 2.5 m/s on CW Doppler interrogation, which all resolved within 3–12 months during the follow-up (52).

The results from these reports are encouraging and provide evidence that transcatheter closure PDA in preterm infants is feasible with an acceptable complications rate (Table 1) and at least study comparing results with surgical ligation demonstrate that outcomes were better with device closure and complications were less compared to surgical ligation (52). However, none of these reports provide long-term outcomes (especially death or BPD) and the impact on neurodevelopment is unknown.

**Table 1:** Success rate and major complications from transcatheter closure of PDA in preterm infants (43, 49, 50, 52, 60–62).

**Author (ref)**	**Mean weight (grams)**	**No of procedures**	**Success rate**	**Major complications (no of cases)**
Baspinar et al. (62)	1,700 ± 300 g	16	13/16 (81%)	Device embolization (2); Death from cardiac perforation (1)
Zahn et al. (43, 61)	1,250 g (range: 755–2,380 g)	23	20/23 (87%)	Transient descending aortic obstruction (1); LPA stenosis needing stenting (1)
Morville et al. (60)	<2,500 g; 17 under 1,200 g	34	32/34 (94%)	Death from hemopericardium (1); LPA obstruction requiring surgical device removal (1)
Sathanandam et al. (50)	<2,000 g	82	80/82 (98%)	Death from inferior vena cava laceration (1); Severe LPA stenosis needing device retrieval (1); Pericardial effusion needing drainage (1)
Rodriguez Ogando et al. (49)	1,260 g (range: 1,000–1,980 g)	27	27 (100%)	No major complications reported
Regan et al. (52)	1,200 g (range: 1,025–1,700 g)	64	63/64 (98%)	Severe LPA stenosis needing device retrieval (2); Aortic arch obstruction needing re-intervention (1);

## Complications

One of the most frequent complications is left pulmonary artery (LPA) obstruction due to protrusion of the device. In a 1,000 g infant, the LPA diameter is ~3 mm whereas the APO retention discs for the 4 and 5 mm-devices are 5.25 and 6.5 mm, respectively. Despite the goal to implant the whole device inside the duct itself, protrusion of the proximal disk may occur especially in patients with a large duct and restriction at the pulmonary end. In cases with significant LPA stenosis (max velocity >2 m/s on continuous wave (CW) Doppler interrogation) diagnosed before the device is released it is recommended to reposition it or to change it for a shorter and/or a smaller device. Significant LPA stenosis has been described with AVP II requiring LPA stenting (61). When the LPA stenosis is mild, the device can sometimes be released as this will resolve in most of the cases during follow-up (49).

**Figure 4:**
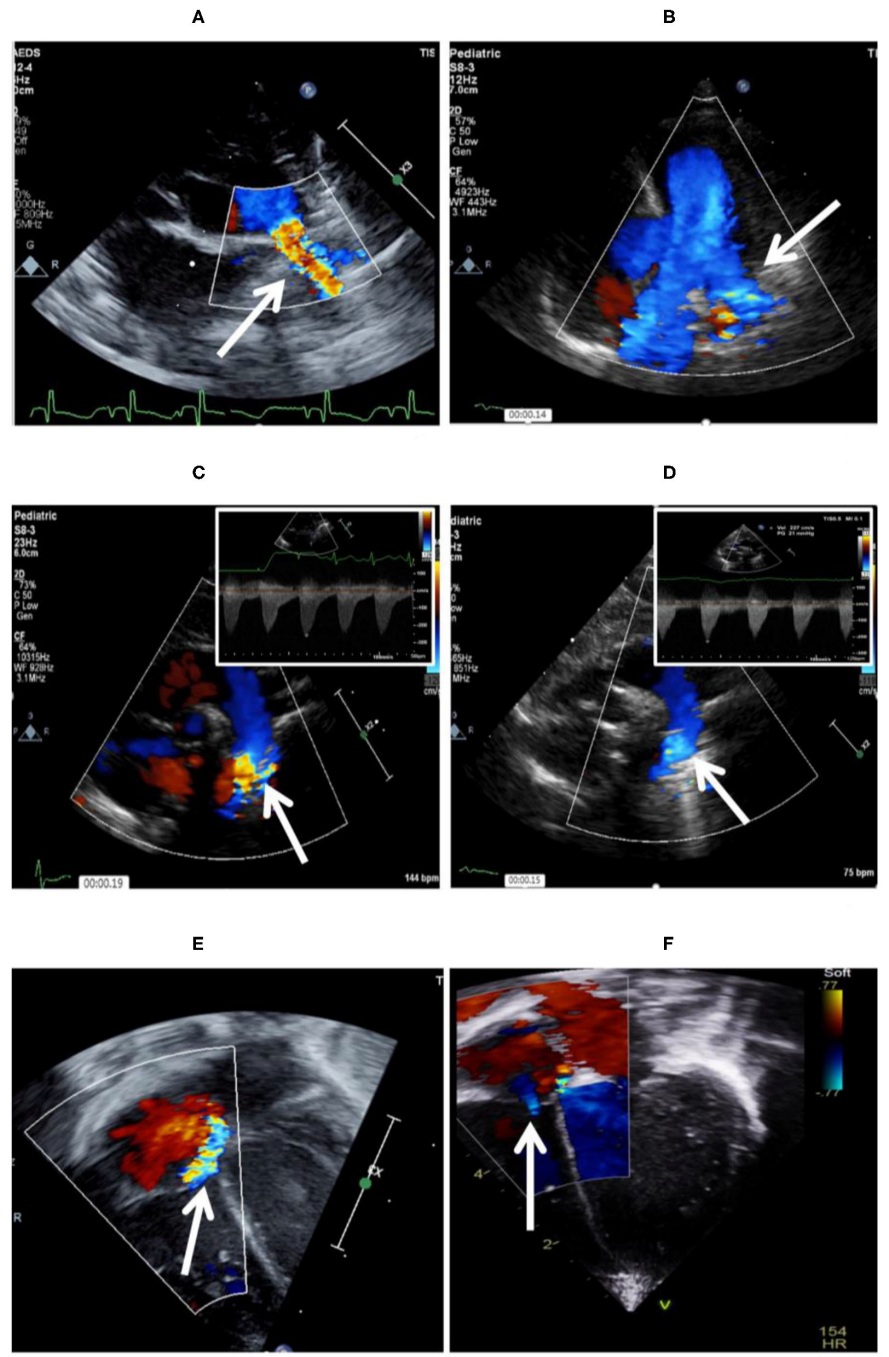
Complications encountered after PDA device closure. **(A)** Shows LPA origin stenosis following percutaneous closure, which is fully resolved 5 months after follow-up **(B)**. **(C)** Mild aortic coarctation after device deployment, which is fully resolved after 11 months of follow-up **(D)**. **(E)** Tricuspid valve trauma noted after successful PDA device closure. **(F)** Tricuspid regurgitation is trivial 12 months after the procedure.

Coarctation of the aorta related to the device is an uncommon complication. It is often diagnosed with transthoracic echocardiography (TTE) during implantation of the device and repositioning is required in such cases. Similar to LPA stenosis, mild coarctation may resolve with patient's growth. However, significant coarctation may develop, necessitating surgical repair (52) (Figure 4).

Device embolization has also been reported during, immediately after and up to 9 days post procedure (48, 49, 63), usually in patients over 1,200 g with a large PDA. The device can be retrieved percutaneously in most cases through a 4Fr or 5Fr sheath (52).

Tricuspid valve injury has been reported and it is often due to chordae rupture from the catheter when the tricuspid valve is crossed.

Rupture and/or dissection of inferior vena cava has been reported as a fatal complication (50). Position of the wire during the femoral vein access should be checked by fluoroscopy before advancing any sheath to minimize this potentially life-threatening complication.

Morville et al. reported fatal cardiac perforation in a 680 g patient. During the procedure the right ventricle was perforated by a 4Fr catheter over a 0.018 Terumo wire, creating a hemopericardium (64). Use of 0.014 soft coronary wire should be advocated in the smallest patients.

There is an existing gap in knowledge in comparing the immediate post-PDA closure complication rates among those undergoing surgical ligation vs. transcatheter occlusion and at this stage no studies have been published comparing such data.

## Future Direction

Transcatheter closure of PDA intuitively appear to be advantageous in ELBW infants. A refined an echocardiographic guided antegrade femoral venous approach, which can be performed at the bedside with little or no contrast and minimal radiation exposure has been developed. Early results from the US ADO II-AS multicenter clinical trial are encouraging. We are cautiously optimistic that ongoing modifications in device design and techniques may ultimately make this a routine bedside procedure performed in the neonatal intensive care unit. However, carefully designed prospective randomized trials studying short- and long-term outcomes will be necessary to determine whether this novel therapy should be the new standard of care. Whether early transcutaneous closure of PDA in ELBW infants will result in improved short- and long- term outcomes, less BPD, improved neurodevelopment, or better long term renal function remains to be seen.

## Conclusion

Transcatheter PDA closure in preterm ELBW infants is technically feasible with high PDA occlusion success rates and acceptable complication rates. The current technique and available devices apply for most of the population, including extremely low birth weight infants. Although follow up studies report excellent short and medium term outcomes, they need to be further tested in the prospective, well-designed and probably randomized controlled trials comparing the results and outcomes of this technique vs. current treatment strategies including medical treatment. This innovative technique is being adopted in increasing number of centers across the globe, however further experience is needed. There is an urgent need for multicenter studies and registries to better clarify the results, optimal timing for this procedure, and to study the short-term and long-term outcomes before this can be considered as an alternative first line therapy, when PDA closure is required in ELBW infants.

## Author Contributions

YS and AF conceptualized the idea and prepared the draft manuscript. All authors contributed to the manuscript. All authors contributed to the article and approved the submitted version.

## Conflict of Interest

AF is consultant and proctor for Abbott and for Occlutech. The remaining authors declare that the research was conducted in the absence of any commercial or financial relationships that could be construed as a potential conflict of interest.
